# Mie Resonance-Modulated Spatial Distributions of Photogenerated Carriers in Poly(3-hexylthiophene-2,5-diyl)/Silicon Nanopillars

**DOI:** 10.1038/srep29472

**Published:** 2016-07-08

**Authors:** Eunah Kim, Yunae Cho, Ahrum Sohn, Heewon Hwang, Y. U. Lee, Kyungkon Kim, Hyeong-Ho Park, Joondong Kim, J. W. Wu, Dong-Wook Kim

**Affiliations:** 1 Department of Physics, Ewha Womans University, Seoul 120-750, Korea; 2 Department of Chemistry and Nano Science, Ewha Womans University, Seoul 120-750, Korea; 3 Applied Device and Material Lab., Device Technology Division, Korea Advanced Nanofab Center (KANC), Suwon 443-270, Korea; 4 Department of Electrical Engineering, Incheon National University, Incheon 406-772, Korea

## Abstract

Organic/silicon hybrid solar cells have great potential as low-cost, high-efficiency photovoltaic devices. The superior light trapping capability, mediated by the optical resonances, of the organic/silicon hybrid nanostructure-based cells enhances their optical performance. In this work, we fabricated Si nanopillar (NP) arrays coated with organic semiconductor, poly(3-hexylthiophene-2,5-diyl), layers. Experimental and calculated optical properties of the samples showed that Mie-resonance strongly concentrated incoming light in the NPs. Spatial mapping of surface photovoltage, *i*.*e*., changes in the surface potential under illumination, using Kelvin probe force microscopy enabled us to visualize the local behavior of the photogenerated carriers in our samples. Under red light, surface photovoltage was much larger (63 meV) on the top surface of a NP than on a planar sample (13 meV), which demonstrated that the confined light in the NPs produced numerous carriers within the NPs. Since the silicon NPs provide pathways for efficient carrier transportation, high collection probability of the photogenerated carriers near the NPs can be expected. This suggests that the optical resonance in organic/silicon hybrid nanostructures benefits not only broad-band light trapping but also efficient carrier collection.

As active materials for photovoltaic (PV) applications organic semiconductors have many advantages, including strong light absorption over a broad wavelength range, adjustable energy band alignment, and competitive fabrication costs^1^,^2^,^3^,^4^,^5^,^6^,^7^,^8^,^9^,^10^,^11^,^12^,^13^,^14^. Despite such advantages, the extremely short exciton diffusion lengths in organic materials (usually <20 nm) limit efficient exciton dissociation to carriers at the junction interface and collection of carriers^1^,^2^. Organic/silicon hybrid (OSH) devices have been proposed to combine the advantages of both organic semiconductors and inorganic silicon (Si). In particular, Si nanostructures, such as nanopillars (NPs), nanowires, and nanoholes, coated with organic layers have attracted significant attention for high-efficiency OSH solar cells. The Si nanostructures can provide pathways for efficient carrier transportation because of the high mobility of Si and the large junction area in such devices can aid efficient exciton dissociation^1^,^2^,^3^,^4^,^5^,^6^,^7^,^8^,^9^,^10^,^11^,^12^,^13^,^14^. Recently, suppression of the surface recombination of nanostructured OSH PV devices enabled notable improvement of their power conversion efficiency^8^,^9^,^10^,^11^,^12^,^13^,^14^.

The optical properties of nanostructure arrays differ considerably from those of their planar counterparts because they have graded refractive indices and because of their multiple scattering, interference, diffraction, and Mie resonance behaviours^15^,^16^,^17^,^18^,^19^,^20^,^21^,^22^,^23^. In particular, Mie resonance can strongly concentrate light of a broad wavelength range near the sample surface^17^,^18^,^19^,^20^,^21^,^22^,^23^. Enhanced fluorescence^20^, Raman^21^, and photoluminescence^22^ intensity from Si NP arrays could be explained by the surface-concentrated light. Such a modified light distribution affects the generation of charge carriers as well as the optical response of the samples^23^. The generation and subsequent redistribution of carriers under illumination changes the surface potential (*V*_*surf*_) of the sample. Thus, the generation of electron-hole pairs and their separation directly determine surface photovoltage (SPV), defined as the difference of *V*_*surf*_ in the dark and under light. Kelvin probe force microscopy (KPFM) enables real space mapping of *V*_*surf*_ with nanoscopic spatial resolution^24^,^25^,^26^.

In this work, we fabricated Si NP arrays coated with a layer of poly(3-hexylthiophene-2,5-diyl) (P3HT) organic semiconductor and studied their SPV characteristics using KPFM. The SPV maps show us how the optical resonance mediated surface concentration of light affects the generation and redistribution of photogenerated carriers in the NPs. We also compare optical simulation results with our experimental data to achieve a better understanding of the SPV behaviours. The results suggest that the spatial distribution of the carriers must be considered carefully to optimize the design of nanostructure based hybrid solar cells.

NP arrays (area: 1 × 1 mm^2^) were fabricated on 4 inch Si wafers (*n*-type, doping concentration: 10^17^ cm^−3^, Silicon Materials) using electron beam lithography and reactive dry etching. The fabrication procedures were identical to those in our earlier work^25^. The scanning electron microscope (SEM) images in Fig. 1a,b show that the diameter, height, and period of the NP arrays were 250, 150, and 1000 nm, respectively. P3HT was spin coated onto the Si wafers at 1200 rpm for 1 min using a stable solution of P3HT in chlorobenzene. From SEM images the thickness of the P3HT layer on a planar Si wafer was estimated to be 40 nm (Fig. 1c). The P3HT layers coated on the Si wafers with the NP arrays were not of uniform thickness: the thickness of the P3HT layer on the flat substrate region around the NPs was 40 nm, comparable to the layer thickness on the planar wafer; however, the thickness of P3HT on top of the NPs was only 10 nm (Fig. 1d).

*V*_*surf*_ of the samples in the dark and under illumination by a red laser (wavelength *λ* = 635 nm) was measured using a KPFM system (XE-100, Park Systems) inside a glove box^24^,^25^. The glove box was purged with N_2_ gas for at least 3 h after loading each sample and the N_2_ atmosphere was maintained throughout the experimental procedures. The laser beam was incident on the sample surface at an angle of 55° to avoid the KPFM head blocking the light. As illustrated in Fig. 2, *V*_*surf*_ is determined by the following equation, *eV*_*surf*_ = (*W*_*tip*_ − *W*_*Si*_) − *ϕ*_*b*_ − *eV*_*P3HT*_, where *e* is electron charge, *W*_*tip*_ is the work function of the KPFM tip, and *W*_*Si*_ is the work function of Si. At the P3HT/Si interface charge transfer occurs and the net charges induce a potential drop across the P3HT layer (*V*_*P3HT*_) and the Si surface (*ϕ*_*b*_)^2^,^3^. This interfacial potential gradient separates the photogenerated charge carriers under illumination and alters *V*_*surf*_, resulting in non-zero SPV (*i*.*e*., SPV = *e*[*V*_*surf*,*light*_ − *V*_*surf*,*dark*_] ≠ 0).

Figure 3a schematically illustrates the four kinds of samples investigated in this work: planar Si wafers and Si NP samples, both with and without a P3HT layer. The reflection spectra of the four samples are presented in Fig. 3b. The experimental spectra were obtained using a micro-spectrophotometer^27^, which enabled us to characterize the small-sized NP samples (area: 1 × 1 mm^2^). The NP sample without P3HT exhibits lower reflection over a broad wavelength range than a bare planar Si wafer even though the surface coverage of the NPs on the sample surface is only 5%. Our widely spaced, short NPs with a period of 1000 nm and height of 150 nm barely caused any variation in the effective refractive index at the surface and showed only small multiple scattering effects^23^. We suggest that the antireflection (AR) behaviour of the NP sample should be attributed to the increase of the scattering and absorption cross sections of the NPs caused by Mie resonance^17^,^18^,^19^,^20^,^21^,^22^. Spinelli *et al*. demonstrated experimentally that the strongly scattered light from their NPs, which had a similar diameter and height as ours, coupled with the substrate and caused the broad-band AR effect^17^. Coating with a P3HT layer drastically decreases the reflection of both the planar and NP samples, because the real part of the refractive index of P3HT (*n*_P3HT_) is smaller than that of Si (*n*_Si_) over the whole measurement wavelength range^7^. Although the reflection of the P3HT coated NP sample is very similar to that of the coated planar sample, the spatial distributions of the incident light in the samples are very distinct. The Mie resonance in the NPs strongly concentrates the light near the surface^17^,^18^,^19^,^20^,^21^,^22^,^23^. Figure 3c depicts the reflection spectra calculated by finite-difference time-domain (FDTD) simulations (Lumerical FDTD Solutions), which reproduce the main features observed in the experimental spectra in Fig. 3b well. In our micro-spectrophotometer, the reflected light was collected by a microscope objective and directed to a UV/vis spectrometer *via* an optical fibre. Incomplete collection of the light and/or spectral distortion in the fibre can cause some quantitative discrepancies between the experimental and calculated reflection values.

Figure 4a,b show the FDTD simulated optical generation rate (*G*) in the P3HT coated planar Si wafers and Si NP array samples under illumination (*λ*: 450, 635, and 850 nm) with an incidence angle of 55°, analogous to the SPV measurement conditions. *G*, representing the number of photogenerated carriers at each point per unit time, is calculated using the equation 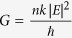, where *n* is the real part of the refractive index, *k* is the imaginary part of the refractive index, and *E* is the electric field^7^. The dielectric functions of P3HT and crystalline Si were taken from ref. 7 and 28. At *λ* = 450 nm, very large *G* values appear in the P3HT layer of both the planar and NP array sample, because P3HT has a high absorption coefficient at such short wavelengths^7^. Interestingly, very large *G* values are also seen in the Si NPs buried underneath the P3HT layer. In the planar sample, the incident light intensity decreases monotonically away from the sample surface. In contrast, in the NP sample the Mie resonance concentrated the light in the NPs and substantially increased the light intensity^17^ and thereby also the *G* values. The photogenerated charge carriers near the P3HT/Si interface can be readily separated and collected by the interfacial potential gradient (Fig. 2). Thus, the surface-concentrated light in the nanostructures can improve the photovoltaic performance of the OSH nanostructured cells.

At *λ* = 635 nm, *G* values are large near the surface of the P3HT layer in the planar sample. In the case of the NP sample with P3HT, the largest *G* values appear in the NPs, while the *G* values in the P3HT layer are relatively small. We attribute the differences between the planar and NP samples to the light confinement in the NPs. At *λ* = 850 nm, *G* in both the planar and NP samples is much smaller than at *λ* = 450 and 635 nm, because the imaginary parts of the refractive indices of P3HT and Si are very small at long wavelengths^7^,^28^. Transverse magnetic mode light was used to obtain the maps in Fig. 4a,b. The spatial maps under illumination of transverse electric mode light (not shown here) also showed significantly enhanced *G* values in the NPs. These results suggest that the optical resonance can substantially increase the number of photogenerated carriers near the NPs. As discussed above, the photogenerated charge carriers near the surface have very high collection probability due to the band bending at the P3HT/Si interface. Therefore, the Si NPs can raise the power conversion efficiency of the OSH nanostructured cells.

Figure 5a,b display surface topography and *V*_*surf*_ maps of a P3HT coated Si NP sample, respectively. In our measurements, a red laser with *λ* = 635 nm was chosen as the light source because the spatial distribution of photogenerated carriers in the NP sample may be very different from that in its planar counterpart, as revealed in the FDTD simulations (Fig. 4b). The exact surface profile at the edge of the NPs could not be obtained because of the tip-sample convolution^26^,^29^. Similarly, *V*_*surf*_ at the NP edges could not be precisely determined because of this geometric artefact^29^. Apparently larger diameter in the AFM image (Fig. 5a) is the manifestation of such artefacts. The *V*_*surf*_ values representing the characteristics at the top surface of the NPs were estimated only at the central region of the NP top surface (indicated by black circles in Fig. 5a,b). To estimate the *V*_*surf*_ value of the flat region around the NP, two rectangular areas above and below the NP, not including the artefact-related *V*_*surf*_ data around the NP, were chosen (see Fig. S1). For comparison, the surface topography and *V*_*surf*_ maps of a P3HT coated planar Si sample were also studied, as shown in Fig. 5c,d. The difference of *V*_*surf*_ in the dark and under light illumination (*i*.*e*., SPV = *e*[*V*_*surf*,*light*_ − *V*_*surf*,*dark*_]) is larger at the top surface of the NP (63 meV) than in the flat region around the NP (40 meV), as shown in Fig. 5e (see Fig. S2). The SPV value of the P3HT coated planar Si wafer (13 meV) is smaller than that of the coated NP sample. The P3HT layer induces band bending at the P3HT/Si interface (as illustrated in Fig. 2), resulting in non-zero SPV values for the corresponding P3HT coated samples. It should be noted that the SPV in the flat region around the NP is larger than that for the planar wafer coated with P3HT. Because the thickness of the P3HT layer is similar in both coated samples, the interfacial band bending in the dark is expected to be similar. Thus, differences in the number of photogenerated charges should cause distinct SPV values. As shown in Fig. 4a,b, the *G* value in the P3HT layer of the flat region around the Si NP is smaller than that in the P3HT layer on the planar Si wafer. This suggests that the concentrated photogenerated charges in the Si NPs diffuse into the neighbouring flat region around the NPs and increase the SPV values.

The SPV behaviour in the P3HT NP sample was determined by the generation, separation, transfer, and transport of charge carriers, as illustrated in Fig. 6^ ^2,3. Because the energy of the red light is larger than the bandgap energies of both Si and P3HT, the incoming light generates charge carriers in both Si and P3HT. When electron-hole pairs are generated in Si, the electrons and holes move toward the Si bulk and P3HT/Si interface, respectively, because of interfacial potential gradient. This charge movement decreases *ϕ*_*b*_ and *V*_*P3HT*_. As a result, the SPV will be positive because SPV is given by *e*(*V*_*surf,light*_ − *eV*_*surf,dark*_) = (*ϕ*_*b,dark*_ − *ϕ*_*b,light*_) + *e*(*V*_*P3HT,dark*_ − *V*_*P3HT,light*_). When the incident light generates excitons in the P3HT layer, some of the excitons reach the P3HT/Si interface and are dissociated there by the electric field at the junction. After exciton dissociation, the holes will move toward the P3HT layer, and the electrons toward the Si bulk; alternatively, the electrons may fill trap states at the interface (indicated as short bars at the P3HT/Si interface in Fig. 6). Except for such trapped charges at the interface, all the photogenerated charges cause the SPV to be positive. Enhanced carrier generation in the Si NPs, clearly shown in Fig. 4a,b, readily explains the reason why the NP sample exhibits larger SPVs than its planar counterpart. The highly concentrated charges generated in the NPs diffuse to neighbouring regions, which raises the SPV in the flat region around the NP, as shown in Fig. 5b,c. In many previous studies, much attention was paid to the electrical benefits of OSH nanostructure based PV devices, such as their enlarged junction area and efficient transport paths^1^,^2^,^3^,^4^,^5^,^6^,^7^,^8^,^9^,^10^,^11^,^12^,^13^,^14^. The SPV maps in this work suggest that the concentrated photogenerated charges can further enhance the PV performance of OSH nanostructure devices.

We fabricated P3HT coated Si NP array samples and compared their SPV characteristics with those of P3HT coated planar Si samples. FDTD simulations and experimental results showed that the NP sample exhibited Mie resonance in the visible wavelength range. The optical resonance strongly confined the incoming light in the NPs, modulating the spatial distribution of photogenerated carriers. Nanoscopic SPV characterization using KPFM clearly visualized the local modulation of the generation and transport of photocarriers in the P3HT/Si NP samples. The SPV values of the NP sample (63 meV) were much larger than those of the planar sample (13 meV). SPV maps also showed that the SPV of the flat region around the NP (40 meV) was larger than that of the planar sample. This suggests that the optical resonance as well as the enlarged junction area in the OSH nanostructured PV devices enables efficient carrier collection.

## Methods

### SPV measurements

First, we measured the topography and *V*_*surf*_ in the dark (*V*_*surf*,*dark*_) from a specified area on the sample surface. Without moving the tip position, we measured the topography and *V*_*surf*_ under illumination (*V*_*surf*,*light*_). We identified the same region from the scanned images using relative coordinates from a notable topographic feature (*e*.*g*., NP). The SPV could be obtained from the *V*_*surf*,*dark*_ and *V*_*surf*,*light*_ maps.

### Optical simulations

FDTD simulations were carried out to obtain reflection spectra and the spatial distribution of the optical generation rate using Lumerical FDTD Solutions version 8.7.4. Periodic boundary conditions and perfectly matched layers were used at the side walls and at the top and bottom of the simulated unit cell, respectively. A linearly polarized plane wave light source was used and the wavelength range was from 400 to 900 nm. For the reflection calculation, the light was normally incident on the sample surface. For the optical generation rate estimation the incidence angle was 55° for direct comparison with the SPV maps to reveal the spatial distribution of the photogenerated charges.

## Additional Information

**How to cite this article**: Kim, E. *et al*. Mie Resonance-Modulated Spatial Distributions of Photogenerated Carriers in Poly(3-hexylthiophene-2,5-diyl)/Silicon Nanopillars. *Sci. Rep*. **6**, 29472; doi: 10.1038/srep29472 (2016).

## Supplementary Material

Supplementary Information

## Figures and Tables

**Figure 1:**
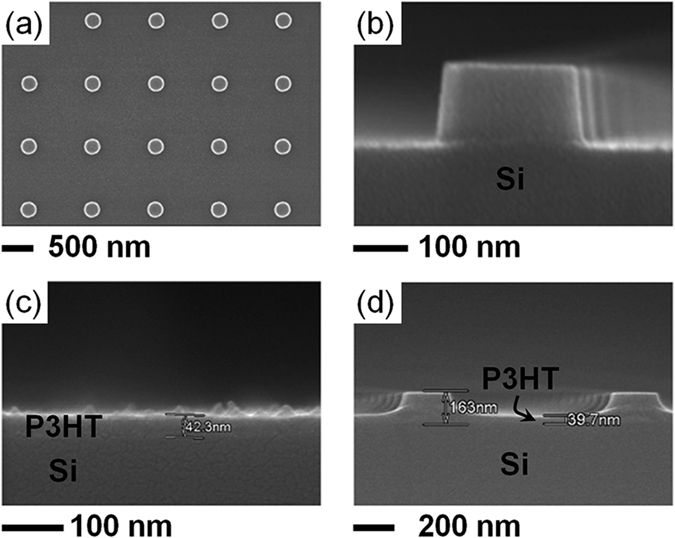
Top down (**a**) and cross sectional SEM images of bare Si NP array (**b**), planar Si wafer (**c**), and NP array sample coated with P3HT (**d**). The thickness of the P3HT layer on the planar wafer, on the flat region around the NPs, and on top of the NPs was 40, 40, and 10 nm, respectively.

**Figure 2:**
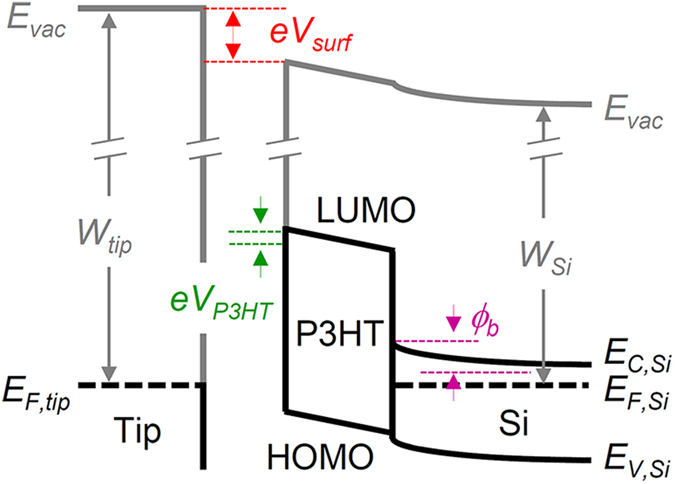
Schematic band diagrams illustrating the surface potential (*V*_*surf*_) measurement of a P3HT coated Si sample using KPFM. *E*_*vac*_ is the vacuum level, *E*_*F,tip*_ is the Fermi level of the tip, and *W*_*tip*_ is the work function of the tip. LUMO and HOMO refer to the lowest unoccupied molecular orbital and highest occupied molecular orbital of the P3HT layer, respectively. *E*_*C,Si*_, *E*_*F,Si*_, *E*_*V,Si*_, and *W*_*Si*_ are the conduction band edge, Fermi level, valence band edge, and work function of Si, respectively. *ϕ*_*b*_ represents the surface band bending at the Si surface and *V*_*P3HT*_ is the potential drop across the P3HT layer.

**Figure 3:**
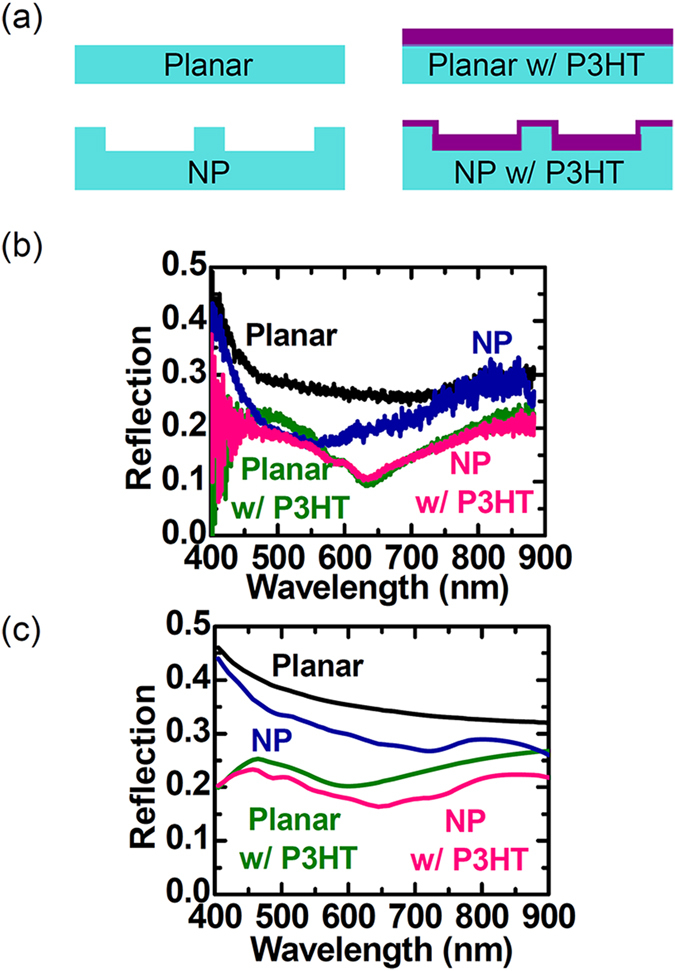
(**a**) Schematic illustrations of the four samples: planar Si wafers and Si NP arrays, both with and without a P3HT layer. (**b**) Experimental and (**c**) simulated optical reflection spectra of the four samples.

**Figure 4:**
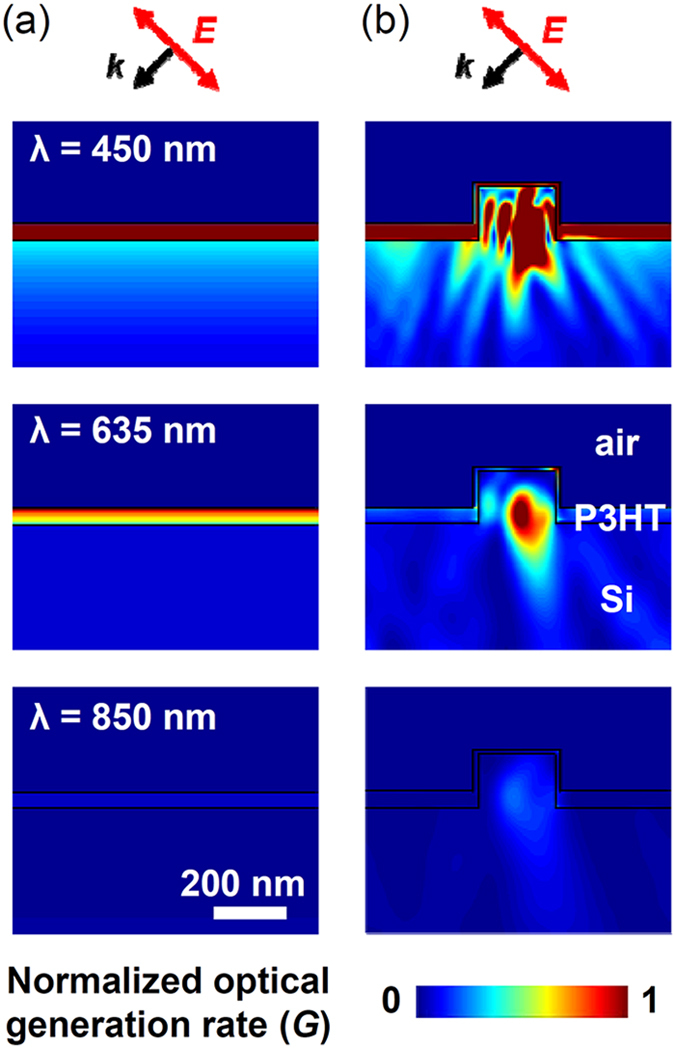
FDTD calculated cross sectional distribution of the optical generation rate (*G*) of (**a**) planar Si wafers and (**b**) Si NP array samples coated with P3HT. The wavelengths of the illumination light were 450, 635, and 850 nm. The incident light was linearly polarized and the incidence angle was 55°, as depicted by the wave vector (*k*) and the electric field (*E*) above the maps. All *G* values are normalized by the largest value that appears in the plots.

**Figure 5:**
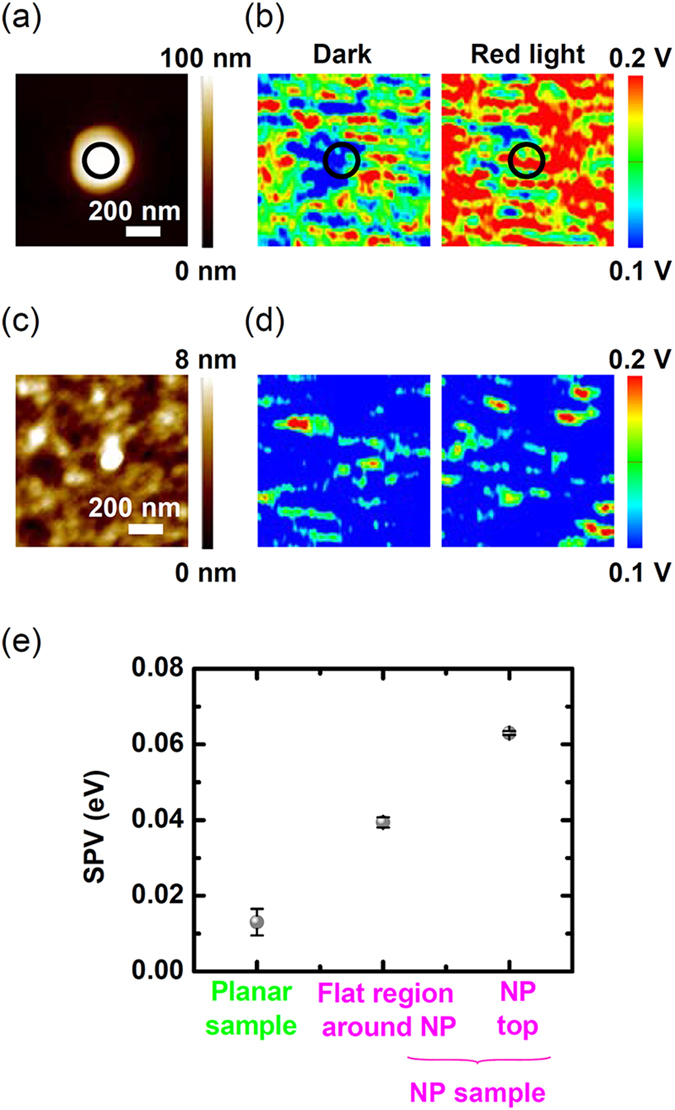
(**a**) Surface topography and (**b**) surface potential (*V*_*surf*_) values of a P3HT coated NP sample in the dark and under light illumination. (**c**) Surface topography and (**d**) *V*_*surf*_ values of a P3HT coated planar Si wafer in the dark and under light illumination. (**e**) SPV values of a P3HT coated planar Si wafer, of a flat region between NPs, and at the top of a NP in a P3HT coated NP sample.

**Figure 6:**
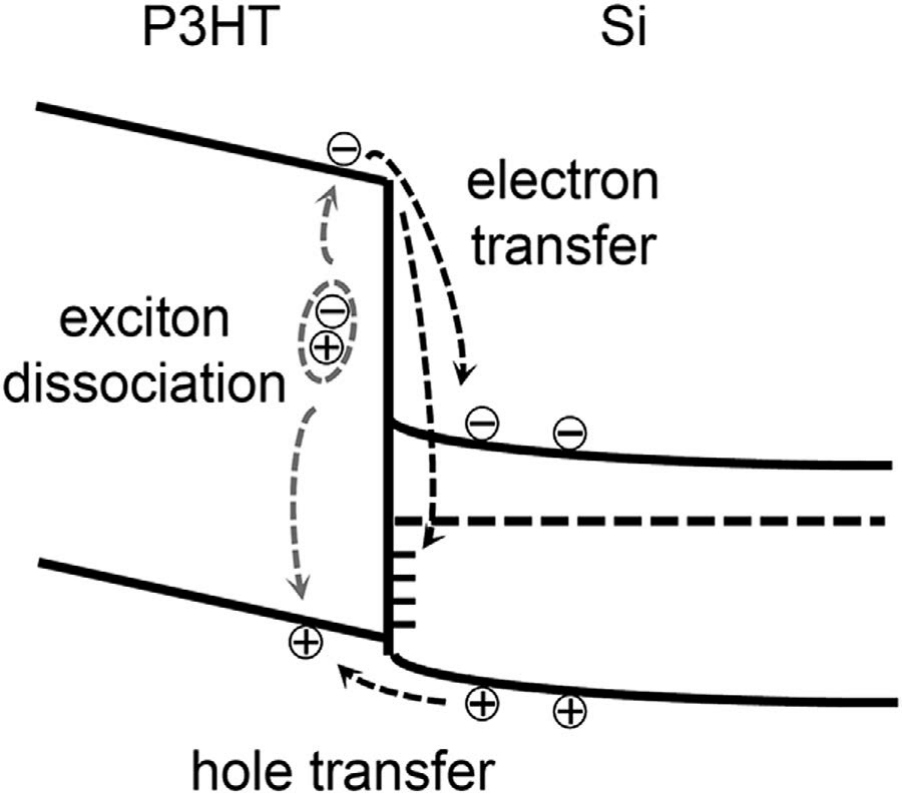
Band profiles and schematic diagrams illustrating photo-excitation and carrier separation processes at the P3HT/Si interface under illumination. Short bars at the interface represent trap states.
