# Supplementary material for: Mie Resonance-Modulated Spatial Distributions of Photogenerated Carriers in Poly(3-hexylthiophene-2,5-diyl)/Silicon Nanopillars
Source: Sci Rep. 2016 Jul 8;6:29472. doi: 10.1038/srep29472 (PMC4937449; doi:10.1038/srep29472)
Supplement: Supplementary Information [file srep29472-s1.pdf]

Supplementary Information for

Mie Resonance-Modulated Spatial Distributions of Photogenerated Carriers in Poly(3-hexylthiophene-2,5-diyl)/Silicon Nanopillars

Eunah Kim,[1] Yunae Cho,[1] Ahrum Sohn,[1] Heewon Hwang,[2] Y. U. Lee,[1] Kyungkon Kim,[2] Hyeong-Ho Park,[3] Joondong Kim,[4] J. W. Wu,[1] and Dong-Wook Kim[1,*]

[1] Department of Physics, Ewha Womans University, Seoul 120750, Korea

[2] Department of Chemistry and Nano Science, Ewha Womans University, Seoul 120-750, Korea

[3] Applied Device and Material Lab., Device Technology Division, Korea Advanced Nanofab Center (KANC), Suwon 443-270, Korea

[4] Department of Electrical Engineering, Incheon National University, Incheon 406-772, Korea

* Correspondence and requests for materials should be addressed to D.K. (email: dwkim@ewha.ac.kr)

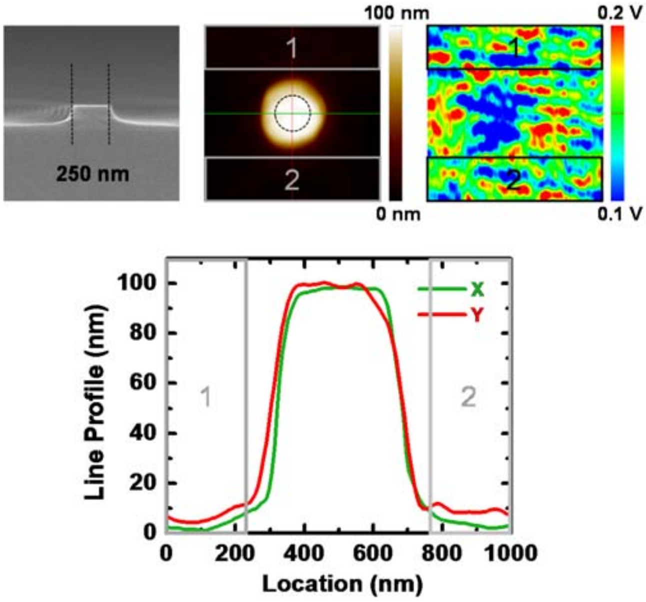

Figure S1: Scanning electron microscopy and atomic force microscopy (AFM) images of a Si NP at the same scale. The dashed lines in the images indicate the NP edges. The rectangular areas 1 and 2 in the contact potential difference map were used to estimate the SPV value at the 'flat region around the NP'. The tip-sample convolution artefact is clearly seen in the line profiles of the green and red lines in the AFM image.

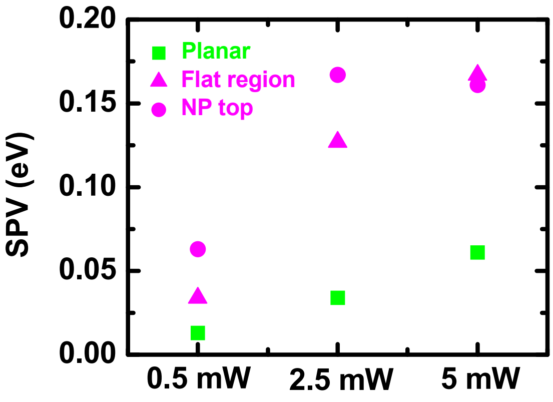

Figure S2: SPV data obtained at three different laser power, adjusted by neutral density filters. 'Planar', 'Flat region', and 'NP top' indicate the P3HT-coated planar Si wafer, the flat region around the NPs in the P3HT-coated Si NP sample, and the NP top of the P3HT-coated Si NP sample, respectively.
